# Coordination of innate immune responses by connexins

**DOI:** 10.3389/fimmu.2025.1594015

**Published:** 2025-05-22

**Authors:** Qirou Wu, Tiejun Zhao, Pinglong Xu

**Affiliations:** ^1^ Key Laboratory of Novel Targets and Drug Study for Neural Repair of Zhejiang Province, School of Medicine, Hangzhou City University, Hangzhou, China; ^2^ Institute of Intelligent Medicine, ZJU-Hangzhou Global Scientific and Technological Innovation Center, Hangzhou, China; ^3^ MOE Laboratory of Biosystems Homeostasis and Protection, Zhejiang Provincial Key Laboratory for Cancer Molecular Cell Biology, Life Sciences Institute, Zhejiang University, Hangzhou, China

**Keywords:** innate immunity, intercellular communication, connexins, gap junctions, signaling molecules, mitochondria transfer, viral infection, inflammation

## Abstract

Innate immunity comprises intricate cellular and tissue responses critical for host defense and tissue homeostasis. Intercellular communication is central to these responses and significantly influences infection, inflammatory disorders, and cancer. Connexins form hemichannels, gap junctions, and connexosomes to mediate signaling molecule transfer, including nucleotide derivatives, ions, antigens, and mitochondria, which occur between adjacent cells or between cells and their microenvironments. By modulating intercellular communication, connexins regulate various immune cell functions and contribute significantly to the coordination of innate immunity. This review summarizes recent insights into connexin-mediated innate immune networks and their implications in pathological contexts such as viral infections, inflammation, and tumorigenesis. Additionally, we discuss targeting connexins as an emerging pharmacological strategy for clinical intervention.

## 1 Introduction

The innate immune system consists of physical barriers, chemical barriers, and cellular components that constitute the first line of defense of our bodies. Among these cellular components, phagocytic cells (such as neutrophils and macrophages), dendritic cells (DCs), natural killer (NK) cells, and a substantial set of non-immune somatic cells operate in an integrated manner to detect and neutralize a diverse array of harmful stimuli (1). This process is generally initiated by the recognition of pathogen-associated molecular patterns (PAMPs) and damage-associated molecular patterns (DAMPs) through pattern recognition receptors (PRRs) (2), which trigger rapid and robust innate immune responses. These responses include the activation of inflammatory pathways, the recruitment of immune cells, and the clearance of pathogens and cellular debris while, at the same time, priming the adaptive immune system (3). Given its pivotal role in numerous diseases ranging from infectious diseases and chronic inflammation to organ fibrosis, neurodegenerative disorders, and cancer, the innate immune system has emerged as a critical therapeutic target (4, 5). The therapeutic potential drives intense investigation into their regulatory mechanisms, where intercellular communication emerges as a vital regulatory factor (6).

**Figure 1:**
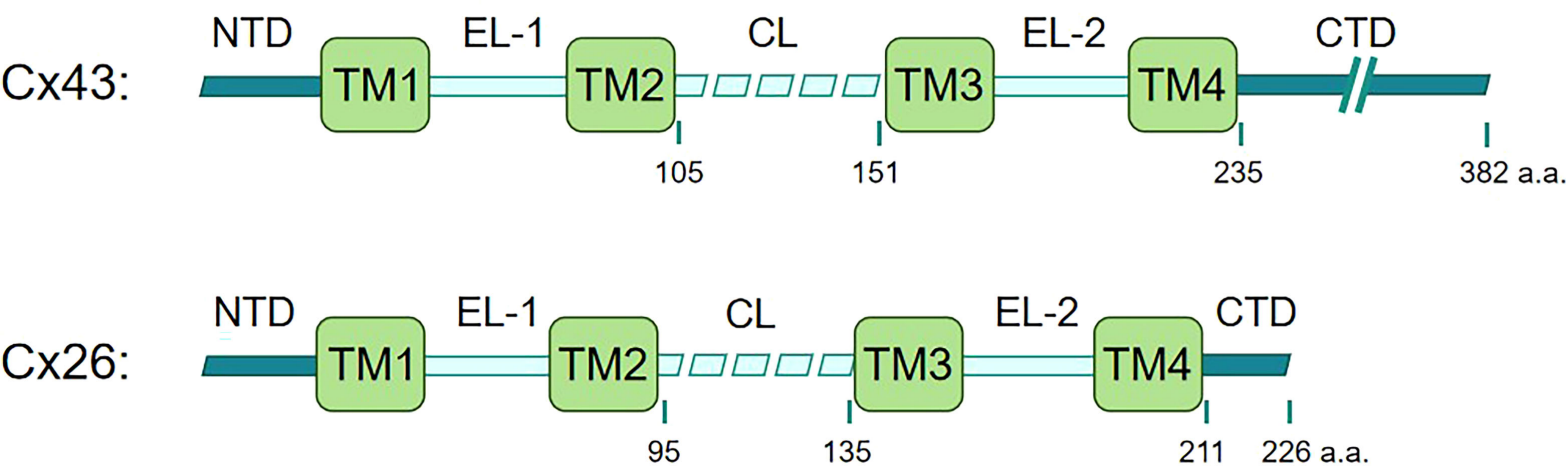
Linear maps of Cx43 and Cx26. Connexins share a similar motif structure, featuring four transmembrane domains (TM1, TM2, TM3, TM4), two extracellular loops (EL1 and EL2) that are notably conserved among connexins, one cytoplasmic loop (CL), amino-terminal and carboxy-terminal domains (NTD and CTD) within the cytoplasmic region (7, 11). The size variations of Cx43 and Cx26 mainly derive from differences in the C-terminal tail.

Recent studies have highlighted the importance of connexins in the innate immune system. Connexins, a family of 21 highly homologous transmembrane members in humans, are best known for their ability to form functional hemichannels and gap junctions. Connexins are named based on their molecular weight. For example, connexin 43 (Cx43) has a molecular weight of approximately 43 kDa. All connexins share a conserved structure featuring transmembrane domains, extracellular loops, a cytoplasmic loop, and cytoplasmic N- and C-terminal tails. Size variations mainly derive from differences in the C-terminal tail containing modification-prone sites, leading to functional differences (**Figure 1**). Additionally, connexins display distinct tissue distributions and are restricted to specific cell types, while some connexins, such as Cx43, are broadly expressed (7, 8). Connexins can oligomerize into hexameric structures termed connexons or hemichannels, which facilitate the exchange of molecules like ATP and NAD+ between a cell and its surrounding environment under certain physiological and pathological conditions like inflammation (9). This functionality resembles that of pannexin, which exclusively forms single-membrane channels. By contrast, connexin hemichannel typically docks with another hemichannel to form a gap junction between adjacent cells, which facilitates electrical coupling and the direct transfer of various signaling molecules, including second messengers, ions, and metabolites (8). In addition, connexins have also been recognized for their intriguing role in mitochondrial transfer (10).

The connexin-containing channels critically modulate key innate immune pathways. For instance, hemichannels and gap junctions facilitate cytosolic cyclic GMP-AMP (cGAMP) transfer, amplifying STING-dependent interferon production (12, 13), while connexin-mediated ATP release primes the NOD-like receptor protein-3 (NLRP3) inflammasome activation and IL-1β/IL-18 secretion (14). Additionally, connexins, especially the most well-documented Cx43, have been reported to regulate innate immune cell functions, including antigen presentation (15), macrophage polarization (16, 17), and phagocytosis (18, 19). Connexins also exert non-channel roles in innate immunity. For instance, Cx43 interacts with poly(ADP-ribose) polymerase 1 (PARP1) to inhibit its nuclear translocation, thereby maintaining NAD+ levels and mitochondrial function, which suppresses excessive inflammation and immune homeostasis (20). Conversely, innate immune signals also influence connexins’ expression, localization, and function in a cell type-dependent manner. For example, upon stimulation with lipopolysaccharide (LPS), the expression and function of Cx43 in macrophages and leukocytes are enhanced (21, 22). In contrast, an adverse effect and degradation of connexins are observed in some parenchymal cells (23). Moreover, cytokines associated with innate immunity, such as IL-1β, can also modulate the opening of Cx43 hemichannels (24).

Despite these advances, only a limited subset of connexins has been extensively surveilled and studied in innate immunity, including Cx43, Cx40, Cx37, Cx26, Cx32, and Cx30.3, particularly in epithelial cells, monocytes, DCs, NK cells, and macrophages, and connexins exhibit some paradoxical roles in disease pathogenesis (19, 25). In the present review, we have examined connexins in transmitting vital innate immune signaling molecules, such as nucleotide derivatives, ions, metabolites, and antigens, to understand their varying contributions that depend on molecular specificity and disease context. Subsequently, the connexin-involved mitochondrial transfer has been addressed. We have also focused on the significance of connexins in regulating immune cell functions and bridging innate and adaptive immunity and discussed the therapeutic advantages of targeting connexins in diseases related to innate immunity.

## 2 Transmitted innate immune molecules via hemichannels and gap junctions

**Figure 2:**
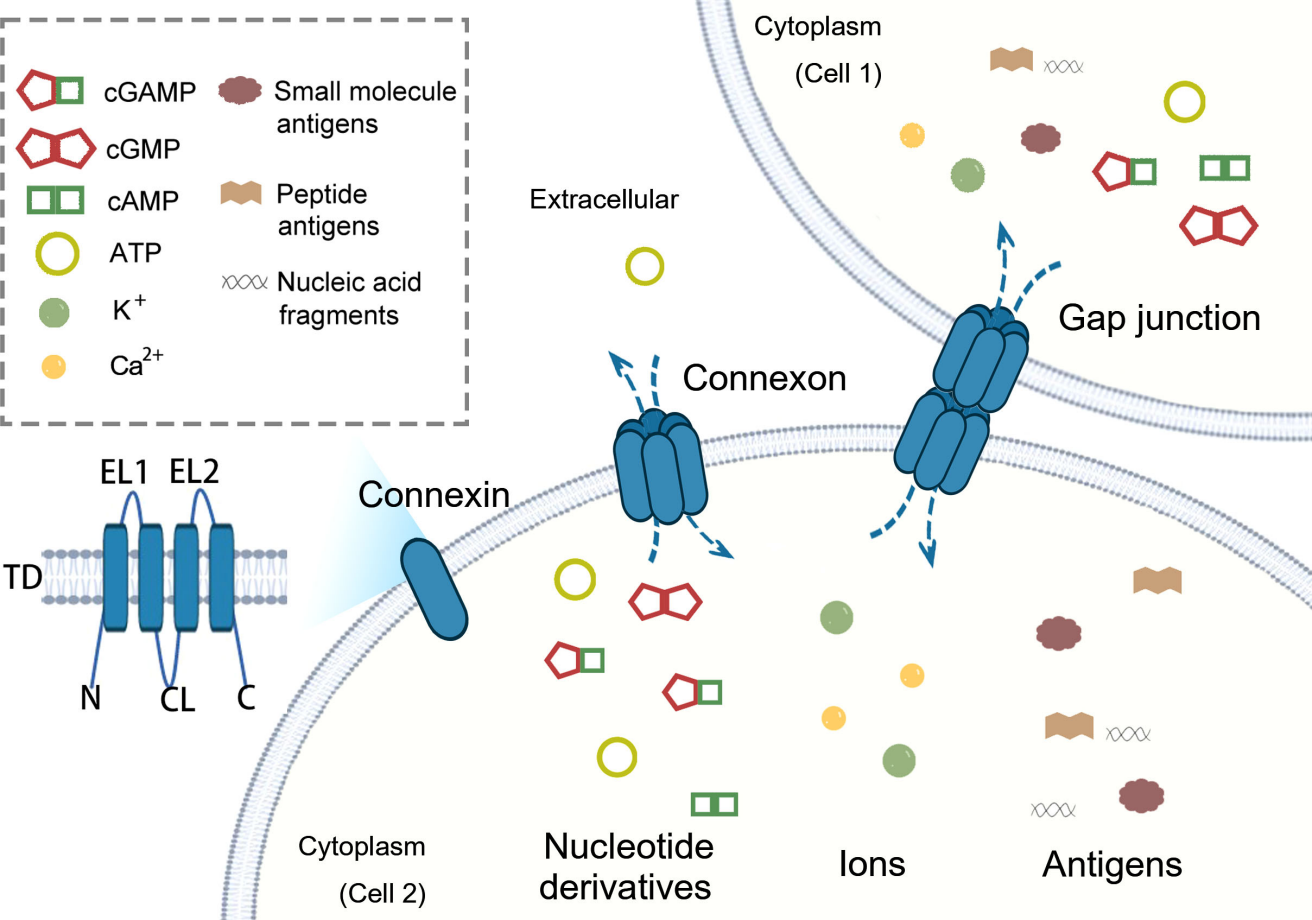
Inter- and extracellular transfer of innate immune signaling molecules through connexin channels. Connexin proteins comprise transmembrane domains, extracellular loops (EL1 and EL2), cytoplasmic loops (CL), and cytoplasmic amino-terminal and carboxy-terminal domains (8). Connexins assemble into hexameric complexes known as hemichannels or connexons. The most widely recognized function of hemichannels is mediating DAMP communications with the extracellular matrix under certain stress conditions. When two hemichannels dock, they form a gap junction channel that connects adjacent cells. Key molecules implicated in immune functions through gap junctions include nucleotide derivatives (such as ATP, cGAMP, cAMP, and cGMP), ions (such as K^+^ and Ca^2+^), and antigens (including small molecule antigens, peptide antigens, and nucleic acid fragments).

Channels formed by connexins have a pore diameter of 1.5–2 nm, allowing the passage of water-soluble molecules with sizes up to approximately one kDa (26). The transmission of innate immune molecules via hemichannels or gap junctions is crucial for a coordinated and swift immune response across multiple cells, particularly among immune cells (27). Under physiological conditions, hemichannels are typically closed or maintain low channel permeability, thereby preserving cellular homeostasis. However, during inflammation, PAMPs can trigger the opening of hemichannels, primarily facilitating the release of ATP and DAMPs (28). When gap junctions form between two cells, the channels are typically open to perform vital physiological functions, and their functionality is intricately linked to the molecules they convey. These molecules include nucleotide derivatives such as cGAMP, ions, and antigens in innate immunity (**Figure 2**). By enabling the rapid and synchronized exchange of signals, gap junctions enhance the efficiency at the tissue level of immune surveillance, inflammation, and clearance of pathogens or damaged cells (27). Clarifying the specific molecules that pass through connexin-mediated channels in diverse contexts contributes to elucidating the functions of connexins in various pathological states and enables us to better intervene in innate immune-related diseases.

### 2.1 Nucleotide derivatives

In multicellular organisms, innate immune defense mechanisms depend on complex cell interactions, mainly mediated by soluble proteins like type I interferons (IFN-Is) (1). However, even in cells with compromised interferon receptors, the activation of immune signaling remains detectable between adjacent cells (6, 29). This persistence is attributed to intracellular communication, particularly the transfer of ATP and second messengers through connexin channels. Among nucleotide derivatives, ATP is the primary molecule for intracellular energy transfer, while cGAMP, cAMP, and cGMP function as critical second messengers. All of these molecules can be transferred through connexin channels.

ATP serves as an important DAMP and purinergic signaling molecule, participating in the regulation of inflammation progression. In damaged or dying cells, ATP can be released through fragmentation of cells, Ca^2+^-dependent exocytosis, or connexin- and pannexin-formed hemichannels, leading to a several hundred-fold increase of extracellular ATP (30). Intriguingly, in innate immune cells, such as macrophages and neutrophils, LPS-induced ATP release was inhibited by deleting the Cx43 gene but not by the pannexins blocker probenecid (31). This observation suggests that connexins and pannexins exhibit distinct patterns of opening and regulation within immune cells despite their structural similarity. Subsequently, extracellular ATP activates the P2X7 receptor on neighboring immune cells, leading to the activation of NLRP3 inflammasome and cleavage of caspase-1, which promotes the secretion of proinflammatory cytokines such as pro-IL-1β and pro-IL-18 (32). Given the role of connexins in ATP efflux, inhibitors of connexin hemichannels can reduce ATP concentrations in the microenvironment, thereby dampening inflammatory signaling. In inflammatory diseases, specific hemichannel inhibitors, such as the Cx43 mimetic peptides TAT-GAP19 and Peptide 5, have demonstrated protective effects in conditions like liver fibrosis, hepatic ischemia/reperfusion injury, and lethal microbial infections (31, 33). Additionally, inhibiting autocrine Cx43-dependent ATP release in macrophages improves the sepsis outcome (34).

cGAMP is a second messenger produced by the enzyme cGAMP synthase (cGAS) in response to the presence of abnormal cytoplasmic double-stranded DNA and functions as a pivotal PAMP and DAMP to produce IFNs and various immune mediators (12, 35). cGAMP can be transferred from producing cells to neighboring cells through gap junctions, which initiates a signaling cascade that amplifies the intensity of innate immune responses (36). This intercellular transmission can occur between epithelial cells, tumor cells, macrophages, and DCs, enhancing antiviral and antitumor responses. The transfer of cGAMP through gap junctions provides a rapid, transcription-independent, horizontal propagation mechanism for activating innate immunity (37). However, the transfer of cGAMP through the gap junction does not always appear beneficial. For example, brain metastatic cancer cells use gap junctions to transfer cGAMP to astrocytes, activating STING signaling and promoting the release of inflammatory cytokines to enhance tumor growth and chemoresistance (12).

cAMP and cGMP, structurally similar to cGAMP, are cyclic nucleotides that can also be transmitted rapidly between adjacent cells through gap junctions (38). However, connexin types affect the permeability of such transmission. For instance, Cx43 is highly expressed in the heart, nervous system, and immune system and plays an important role in facilitating the passage of cAMP and cGMP, which are strongly linked with cardiac and neurological disorders (39, 40). Although limited studies regarding the connexins with cAMP and cGMP transfer in immune cells, their pivotal roles in regulating cell and tissue homeostasis suggest a potential in regulating the innate immune system (41).

### 2.2 Ions

Ion transport is another pivotal function of gap junctions to maintain innate immune homeostasis. The activation of connexin channels enables the free interchange of potassium (K^+^) and calcium (Ca²^+^) ions between adjacent cells, which plays a key role in modulating the NLRP3 inflammasome (42, 43), a process vital in orchestrating the inflammatory response to infection and tissue damage, by activating caspase-1. Caspase-1 also cleaves gasdermin D (GSDMD), leading to pyroptosis, a regulated cell death that contributes to inflammation (44). Ca²^+^ influx is necessary to generate mitochondrial reactive oxygen and activate the NLRP3 inflammasome, and the opening and closing of these hemichannels are controlled by calcium and potassium concentrations (45). NLRP3 inflammasome is closely associated with various connexin-related diseases, including autoimmune disorders, neurodegenerative conditions, and cancers (46). During wound healing, an injury-induced calcium wave increases Ca²^+^ influx, enhancing the activity of nuclear factors of activated T-cells, and these long-term transcriptional and functional responses are regulated by connexins (47). In addition, the deregulated function of connexin channels can mediate abnormal ion flow, leading to cytotoxicity and homeostatic imbalance (48), and subsequently activate innate immune signals.

### 2.3 Antigens

The intercellular transmission of antigenic information from donor to recipient cells can be mediated by gap junctions, which are crucial in regulating immune responses (49). These biomolecules include small-molecule antigens, peptide antigens, and nucleic acid fragments and typically originate from pathogens or foreign substances that activate PRRs and trigger defensive immune responses (50). Connexin-mediated antigen transfer effectively enhances the utilization of antigens and broadens and prolongs the range and duration of immune responses. Early studies on the role of gap junctions in antigen cross-presentation originated in virology. Specifically, disrupting gap junction-mediated intercellular communication reduced immunoglobulin and cytokine expression in mixed lymphocyte cultures, herpes simplex virus (HSV), and human papillomavirus (HPV) suppressed the expression of connexins during latent infections in Vero cells (51). Furthermore, gap junctions facilitate the transfer of intercellular antigen epitopes for presentation on MHC class I molecules, making them accessible for recognition by cytotoxic T lymphocytes (52). Gap junction-mediated antigen cross-presentation is also a key mechanism for activating DCs (53). Additionally, some tissue-resident DCs have limited direct exposure to pathogens; thus, intercellular antigen transfer can effectively overcome this limitation (15). Interestingly, in studies of gap junctions formed by Cx43, peptide segments with molecular weights less than one kDa exhibit excellent passage efficiency (54). By contrast, larger peptide segments restricted by MHC class II tend to be less compatible (55), reflecting the influence of molecular size on the passage efficiency through gap junction channels.

In antitumor immunity, antigen spreading is a common phenomenon. For instance, bacteria-treated melanoma cells can form functional gap junctions with adjacent DCs to transfer antigenic peptides (56). Meanwhile, CCR7-expressing CD103(+)/CD141(+) DCs in melanoma can efficiently transport tumor antigens to activating T cells (57). Connexin-mediated antigen transfer may occur in various physiological and pathological environments, including the thymus, intestines, sites of allergic reactions, lesion areas, and vaccination sites (58). During vaccination, host cells responding to infections or vaccinations transfer antigens to DCs, helping prevent damage to DCs from direct contact with viruses. This transfer compensates for certain subtypes of DCs with limitations in acquiring distal antigens, thereby enhancing specific immune responses against natural infections, tumor development, and vaccine-induced immunity (58). The transfer of antigens at these sites can significantly influence immune outcomes. Therefore, investigating how connexins precisely regulate antigen transfer is important to understanding innate immune modulation toward acquired immunity.

## 3 Connexin-involved mitochondrial transfer

As a dynamic organelle within the cell, the mitochondrion plays a central role in energy production, cellular metabolism, and damage monitoring. Some PRRs on the mitochondrial membrane, such as the RIG-I-like receptors, can sense the presence of pathogens and promote the production of interferons and other inflammatory factors (59). Besides, key components in innate immunity, such as TBK1 kinase, regulate the fusion and fission of mitochondria (60). Intriguingly, recent emerging research has suggested that some cells can export some of their mitochondria and deliver them to recipient cells, such as between tumor cells and macrophages, and is associated with the functional regulation of immune cells (61).

**Figure 3:**
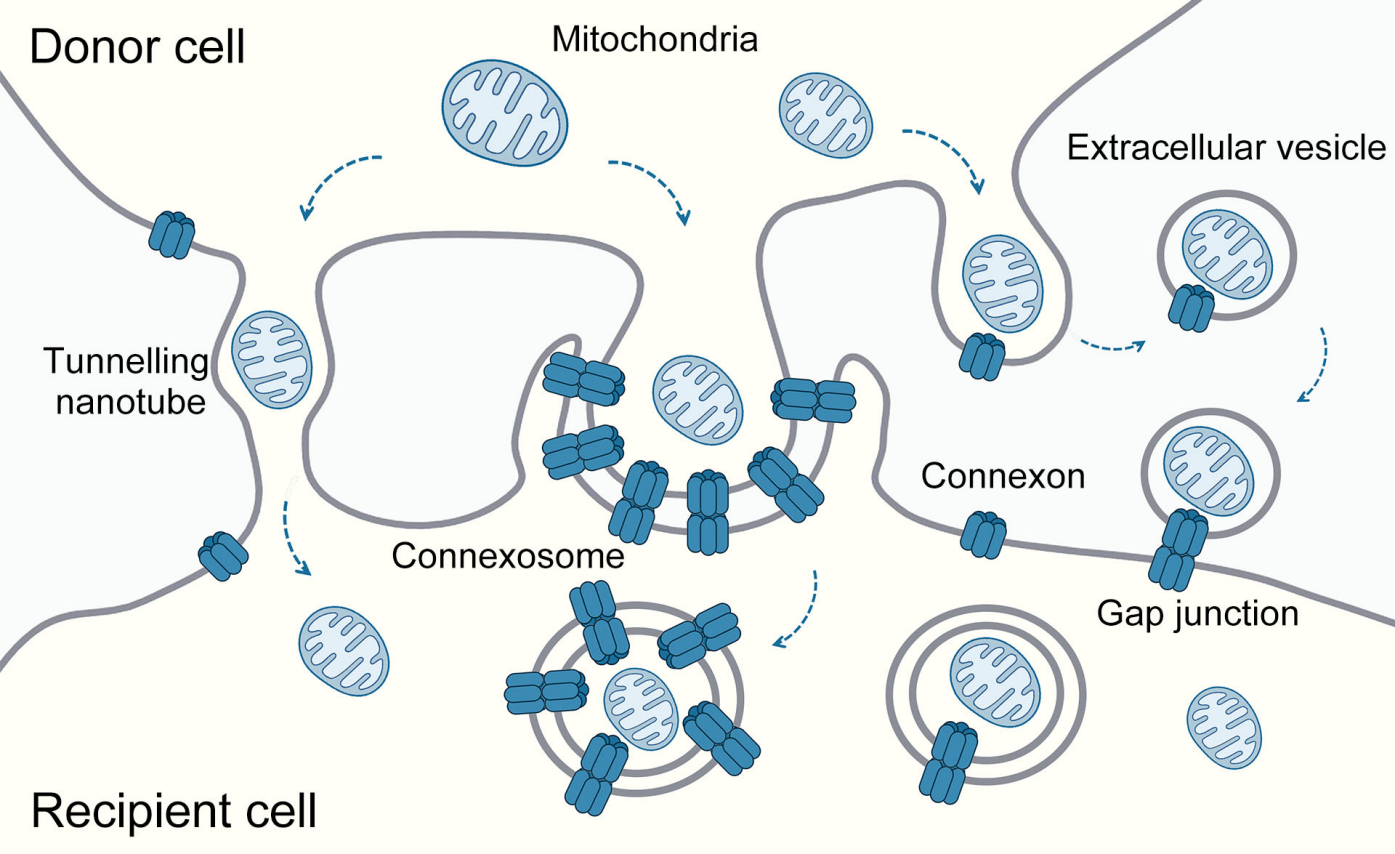
Mechanisms of connexin-involved mitochondrial transfer. Mitochondria can be encapsulated in double-layered vesicles, called connexosomes or annular gap junctions, and is intercellularly transferred by internalizing these connexin-related structures. Mitochondrial transfer between cells can also occur via extracellular vesicles that carry connexons on their surface, enabling fusion with target cells and the transfer of contents directly or through double-layered vesicles. Additionally, tunneling nanotubes, which facilitate the exchange of cellular components, including mitochondria, are modulated by connexins such as Cx43.

Several connexin-involved mechanisms have been reported to facilitate intercellular mitochondria transfer. Tunneling nanotubes represent one of these mechanisms that enhance macrophage phagocytosis (62) and host defense (63), and connexins, such as Cx43, regulate the formation of tunneling nanotubes in some scenarios (64–66). However, since tunneling nanotubes can mediate the sharing of multiple cellular components, it is not easy to attribute their phenotypes solely to mitochondrial transfer. In addition, mitochondria can be incorporated into double-membrane vesicles, called connexosomes or annular gap junctions. The formation of these structures is a consequence of the internalization process of gap junctions (10). Moreover, connexins are also implicated in extracellular vesicle-mediated mitochondrial transfer (61). Extracellular vesicles released from cells expressing connexins may carry connexons on their surface, which can couple with corresponding connexons on the recipient cells, facilitating direct or double-walled vesicle transfer (**Figure 3**). In addition to mitochondria, extracellular vesicles containing connexins can transfer other contents, significantly increasing their potential for engineering intracellular drug delivery (67).

Connexin-involved mitochondrial transfer has also been detected in diseases associated with innate immunity, such as acute injury, infection, and cancer (68). For example, in the mouse model treated with LPS, bone marrow-derived mesenchymal stromal cells (BMSCs) can transfer mitochondria to alveolar epithelial cells in a Cx43-dependent manner, thereby protecting the mice from acute lung injury (69). These findings suggest the multifaceted role of mitochondria in innate immunity and their potential importance in intercellular communication and immune modulation.

Mitochondria play a double-edged role in modulating innate immunity: they initiate inflammatory responses and activation, while excessive oxidative stress may lead to cellular damage and immune dysfunction (59). Beyond the direct transfer of mitochondria, various mitochondrial components, such as mitochondrial DNA (mtDNA), reactive oxygen species (ROS), and specific metabolic byproducts, can also be conveyed between cells through gap junctions (37, 70). The ability of mitochondria to influence immune responses and to be transferred between cells via gap junctions establishes them as pivotal players in the intricate interplay between cellular metabolism and immunity.

## 4 Bridged innate and adaptive immune responses by connexins

**Figure 4:**
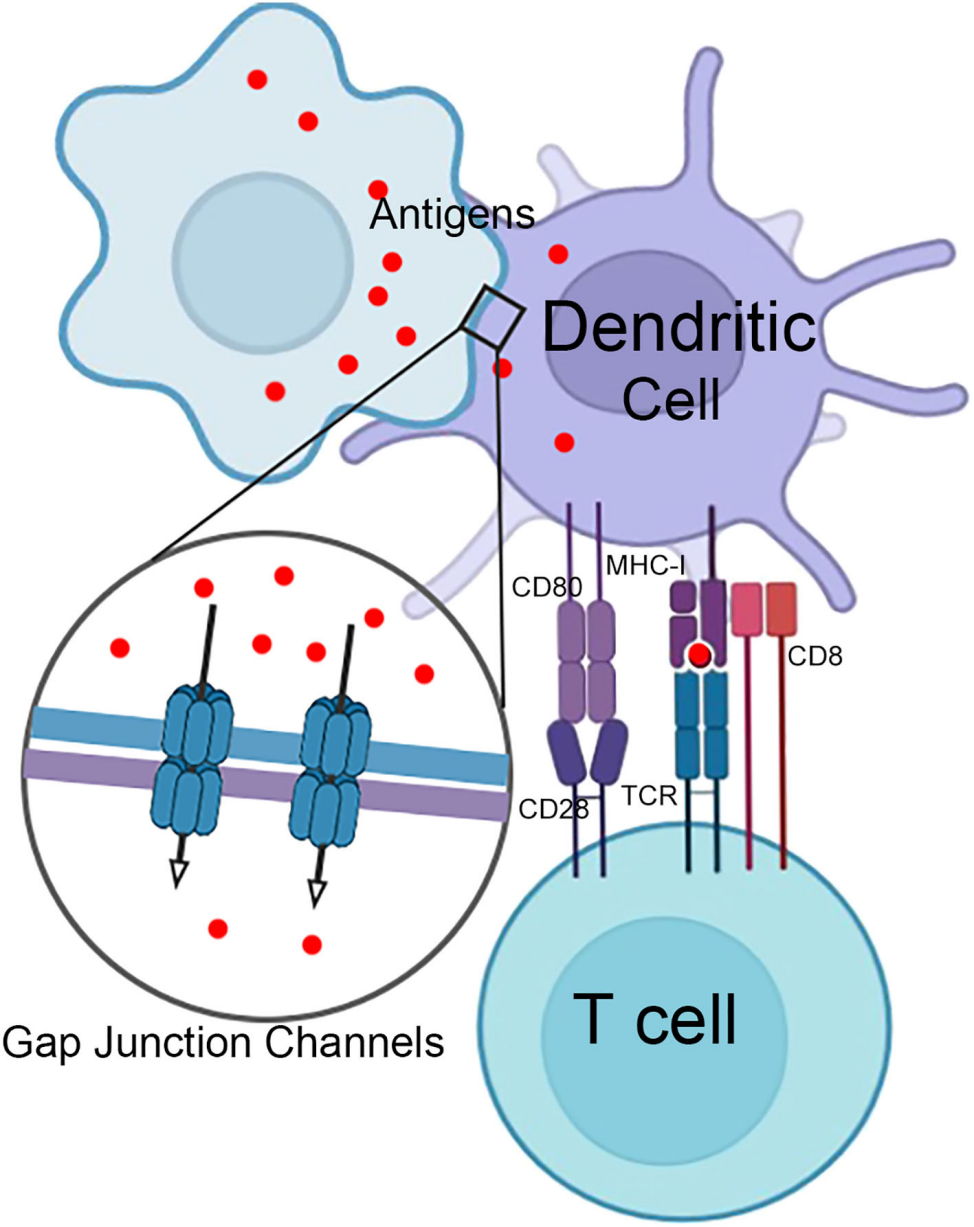
Gap junctions in T cell activation by dendritic cells. Infected or damaged somatic cells and phagocytic cells that have engulfed antigens can transfer antigens to dendritic cells through gap junction channels. This transfer of antigens is essential for initiating an adaptive immune response and subsequently activating T cells.

In addition to mediating intercellular communication, connexins are crucial in regulating immune cell functions, particularly in macrophages and DCs. For instance, Cx43 expression in macrophages is upregulated during inflammation, which enhances their migratory abilities (71). Conversely, macrophages lacking Cx43 exhibit dramatic deficiencies in phagocytosis (18). Connexins facilitate intercellular communication between antigen-presenting cells (APCs) and drive the formation of the immunological synapse between DCs and T cells. These processes modulate antigen presentation, thereby regulating critical T lymphocyte activation (72). DCs are key accessory cells in acquired immunity, playing an essential role in antigen presentation, and isolated lymphocyte populations cannot respond effectively to antigens without them (73). In most instances, DCs fail to elicit an effective immune response when directly confronted with highly invasive and cytotoxic viruses or damaging conditions (58). However, plasmacytoid dendritic cells (pDCs) can tolerate infections from a subset of viruses and produce substantial amounts of IFN (74). Consequently, antigen transfer mediated by gap junctions is of significant importance for the functionality of DCs. Pathogens and innocuous antigens captured by gut-resident macrophages are transferred to migratory DCs via gap junctions, thereby inducing protective immunity (15). In tumors, forming functional gap junction channels between tumor cells and adjacent DCs is crucial for intercellular transport of antigenic peptides (75, 76). Therefore, connexins are important for DCs to perform effective antigen presentation, initiating a specific cytotoxic T lymphocyte response (**Figure 4**).

PRR signaling pathways are another key route for modulating the transition from innate to adaptive immunity, with connexins being a key component in this regulatory network. For example, the intercellular transfer of cGAMP through gap junctions activates the STING in recipient cells (35). Traditionally associated with the innate immune system, STING critically modulates the function of cytotoxic T cells to influence adaptive immunity (77, 78). Similarly, connexin-mediated ATP signaling initiates an “eat me” signal for phagocytosis, a process regulated by immunoglobulin-like domain-containing proteins (IGLDCPs), including CD31, CD46, and CD47, which emit “do not eat me” signals to modulate phagocytic activity (72). The cross-activation between connexins and IGLDCPs also involves the regulation of Tregs to suppress the excessive immune responses through the release of cAMP and other immunosuppressive factors (79). Furthermore, connexins also contribute to trogocytosis, a process for lymphocytes to extract surface molecules from APCs and display them on their membranes. This process promotes antigen presentation and amplification, thereby enhancing adaptive immunity (58, 72).

Several other studies have indicated the involvement of connexins in the immunological synapse. An immunological synapse is a cellular interaction hub established at the interface between two opposing cells, with at least one being an immune cell, facilitating intercellular communication (27). Connexins, notably Cx43, are pivotal in modulating the signaling processes within various immunological synapses. The signaling molecules transmitted through connexin channels are instrumental in the signaling cascades that occur within the immunological synapse and are critical for the activation of T and NK cells, the suppression of immune responses by regulatory T cells, and the elimination of target tumor cells by cytotoxic T lymphocytes or NK cells (80). Considering that the cytoplasmic C-terminus of Cx43 can engage with numerous proteins, it is reasonable to propose that Cx43 serves as a scaffold protein at the IS, coordinating the assembly of various regulatory proteins (80, 81). This notion is reinforced by observations of multiple Cx43-associated proteins relocating to these pivotal cell-cell junctions, indicating a pivotal role of Cx43 connexins in modulating immune cell interactions.

In summary, the connexins are involved in the early stages of pathogen recognition and inflammation, as well as in the later stages of antigen presentation and the generation of immunological memory. Understanding the complex dialogue between innate and adaptive immunity by connexins is essential for developing novel therapeutic approaches to harness multiple phases of the immune response to combat a broad spectrum of health challenges.

## 5 Connexins in the disease pathogenesis

Dysfunctional connexin has been associated with a spectrum of innate immune-related disorders. Such impairments in signaling can result in either insufficient immune responses or overactive inflammation, thereby playing a role in the development of conditions such as viral infections, disruptions in tissue homeostasis, and various forms of cancer (82). However, determining whether the inhibition of gap junctions is beneficial or detrimental remains a contentious issue, with conclusions varying depending on the specific context, and is intimately linked to the types of signaling molecules transmitted through connexin channels. Here, we explore the connections between connexins and innate immunity under various pathological conditions, including viral infections, tissue homeostasis and inflammation, and tumorigenesis.

### 5.1 Microbial infection

In the context of viral infections, the regulatory dynamic of connexins exhibits a complex duality, which varies according to the type of signals transmitted through connexin channels, the nature of the viral proteins, the stage of infection, and the specific cell type involved, all significantly impacting innate immune responses. Various viruses downregulate connexin levels to evade immune surveillance (83). For instance, upon human papillomavirus 16 (HPV16) infection, the E5 protein disrupts gap junctional communication by inhibiting Cx43 phosphorylation (84, 85), and the E6 protein interacts with discs large homolog 1 (Dlg1) to reduce Cx43 levels and relocalizes Cx43 to the cytoplasm (86). These mechanisms are closely linked to the suppression of innate immune responses and cervical cancer progression (87). During human adenovirus type 5 (HAdV-5) infections, the early viral protein E4 open reading frame 1 (E4ORF1) suppresses gap junction gene transcription by activating β-catenin. Additionally, HAdV-5 infection promotes protein kinase B (AKT)-mediated phosphorylation of Cx43 at S373, causing transient gap junction plaque expansion before internalization. These effects lead to arrhythmias in infected hearts (88). Similarly, in human cytomegalovirus (HCMV) infections, the immediate early protein IE1 binds to the C-terminus of Cx43, promoting its degradation via the ubiquitin-proteasome pathway and impairing neuronal migration (89).

Conversely, a few viruses upregulate connexin levels or activities to facilitate viral invasion and disease progression. For example, human T-cell leukemia virus type 1 (HTLV-1) Tax protein and human immunodeficiency virus (HIV)-tat protein enhances mRNA levels of Cx43 (90), and the HIV envelope glycoprotein gp120 increases Cx43 hemichannel activity to transmit ATP, Ca^2+^ and nitric oxide in astrocytes (91). As such, HIV infection enables the transfer of toxic signals from infected astrocytes to neighboring cells, leading to neurocognitive disorders (92, 93). Intriguingly, the application of general gap junction blockers, such as octanol or α-glycyrrhetinic acid (AGA), can reduce apoptosis in cells adjacent to those infected with HIV (92), thus showing a critical role of gap junctions in facilitating HIV-induced neuroinflammation.

Additionally, severe acute respiratory syndrome coronavirus 2 (SARS-CoV-2) exhibits differential regulation of Cx43. The spike protein 1 (S1) downregulates Cx43 expression, while short-term exposure to the spike/membrane protein (S/M) upregulates cell surface Cx43 (94–96), highlighting a distinct role of viral proteins at different stages of infection. Connexin regulation also exhibits isoform specificity, as seen in HPV18-infected normal immortalized keratinocytes (NIKS), from which Cx43 levels decrease while Cx45 was upregulated, accompanied by enhanced gap junction signaling (97). Both downregulated and upregulated connexin protein expression has been observed during bacterial infection (98). Bacterial LPS and serum amyloid A (SAA) upregulate Cx43 and pannexin1 hemichannel in macrophages, constituting a critical endogenous regulatory mechanism of innate immunity that exacerbates progressions like septic pathology (9). Overall, the dual regulation of connexins in microbial infections provides critical insights into host-pathogen interactions and highlights connexins as potential therapeutic targets for anti-infectious strategies.

### 5.2 Tissue homeostasis and inflammation

Under physiological conditions, connexins are crucial mediators in regulating innate immune cell activation and orchestrating the timely resolution of inflammatory responses, thereby maintaining tissue homeostasis. Dysfunction of connexins has been implicated in various pathological conditions across multiple organ systems, including skin, joints, cardiovascular system, and central nervous system (CNS) (99). Specific connexins play distinct roles in innate immune surveillance. For instance, Cx26 and Cx30 are essential for epidermal barrier function, and their deficiency leads to skin pathologies such as keratoderma and ectodermal dysplasia that compromise innate immune defenses (100). Similarly, Cx46 and Cx50 are implicated in developing cataracts due to disrupted lens homeostasis (99). Additionally, in the central nervous system (CNS), microglia form gap junctions to modulate oxidative stress responses, DNA damage repair, and immune surveillance (101), and microglial dysfunction has been implicated in neurodegenerative and demyelinating disorders (102). Beyond their role in specialized immune cells, connexins are essential for hematopoietic homeostasis, with bone marrow gap junctions supporting hematopoietic stem cell function and blood cell regeneration (103). These findings further highlight their critical contribution to tissue integrity and function.

Notably, connexins and innate immunity interplay is bidirectional, as inflammatory mediators dynamically influence connexin expression and function. Innate immune cytokines such as IL-1β and TNF-α regulate connexin activity through multiple signaling pathways. For instance, IL-1β induces Cx43 phosphorylation at Ser368 via mitogen-activated protein kinase (MAPK) (104), which manifests tissue-specificity during inflammatory responses. In brain inflammation, activated microglia release IL-1β and TNF-α, increasing astrocytic Cx43 hemichannel activity (105). The functional significance of this regulation is evident in ischemia, where Cx43 dephosphorylation increases hemichannel activity, and inhibitors like Gap26, GAP19, and Peptide5 mitigate neuronal damage (106). However, modulation of connexin function must precisely balance hemichannel and gap junction activities, as excessive Peptide5 administration exacerbates ischemic injury (107). Connexins also modulate innate immune responses in peripheral tissues. During lung inflammation, cx43-containing gap junctions between alveolar macrophages and epithelial cells coordinate Ca²^+^ wave-mediated intercellular communication, delivering immunosuppressive signals to regulate inflammatory responses (108). Conversely, Cx43 upregulation in various bone cells, including chondrocytes, synovial cells, tendon cells, and ligament cells, contributes to inflammatory pathologies in joint diseases. Therapeutic targeting of Cx43 using siRNA has shown promise in suppressing inflammatory cytokine expression and alleviating collagen-induced arthritis (109). Therefore, these findings suggest connexins as potential therapeutic targets for innate immunity-mediated diseases through their tissue-specific functions and dual roles in hemichannel and gap junction communication.

### 5.3 Tumorigenesis and metastasis

Connexins play multifaceted roles in cancer, orchestrating innate immune responses and the intricate dynamics of the tumor microenvironment. Connexins are frequently suppressed in various cancer types. Pharmacological upregulation of Cx43 using PQ1 in the PyVT spontaneous mammary tumor model substantially inhibits tumor progression (110). In agreement with this observation, Cx32-deficient mice are more susceptible to chemical and radiation-induced liver and lung cancer, while the inhibition of Cx43 increases the incidence of chemically induced lung tumors (111). Moreover, the reconstitution of connexins generally reduces tumorigenesis and promotes a favorable mesenchymal-to-epithelial transition (82, 112). Emerging evidence also suggests that gap junction function can profoundly influence antitumor immunity, particularly through the well-documented “bystander effect.” For instance, sulforaphane upregulates Cx43 expression, thus enhancing chemosensitivity to gemcitabine in pancreatic cancer models (113).

However, recent studies show that connexins also promote invasion, intravasation, extravasation, and metastasis of cancers (111, 114). In brain metastases of breast and lung cancer, PCDH7 promotes Cx43-mediated gap junctions between cancer cells and astrocytes, facilitating cGAMP transfer, which induces IFNα and TNFα production in astrocytes and, thereby, activates STAT1 and NF-κB pathways in cancer cells by a paracrine mechanism, driving tumor growth and conferring chemotherapy resistance (12). Additionally, Cx31 induces the phosphorylation of focal adhesion kinase (FAK) in a spontaneous breast cancer brain metastasis model, which prompts NF-κB activation, tumor cell-astrocyte interaction, and brain metastasis (115). Similarly, mitochondrial transfer influences the proliferation and survival of recipient cancer cells by restoring mitochondrial respiration (116). Cancer cells enhance ATP production and metabolic function by obtaining mitochondria from donor cells in acute myeloid leukemia (AML) models (117) and patient-derived organoid models of glioblastoma stem cells (118). These findings indicate the complex roles of connexins in tumorigenesis and metastasis. In addition to forming gap junctions and hemichannels, connexins have poorly understood non-channel functions in various subcellular compartments. For instance, Cx43 accumulation in the cytoplasm drives cervical cancer advancement, while cytoplasmic Cx26 promotes hypopharyngeal squamous cell carcinoma proliferation (119, 120). Additionally, cytoplasmic Cx32 confers drug resistance in non-small cell lung cancer and hepatocellular carcinoma (121), thus highlighting its therapeutic potential. Additionally, connexins can localize to the nucleus, which is associated with cancer prognosis (122), and to mitochondria, where they induce apoptosis (123). Collectively, therapeutic modulation of connexins in cancer must be carefully balanced, as excessive connexin activity in the tumor microenvironment may promote immune suppression by facilitating the release of immunosuppressive factors.

## 6 Conclusion

Connexins, which form hemichannels, gap junctions, and connexosomes, participate in intercellular mitochondrial transfer and regulate functions of both immune and tissue cells, constituting a vital part of the complex regulatory network in innate immunity. Here, we have highlighted the significance of connexins in mounting a rapid and coordinated defense against pathogens, maintaining physiological homeostasis, and modulating disease progression. Transiting molecules such as cGAMP, Ca²^+^, and antigens through hemichannels and gap junctions enhances immune surveillance and fosters critical crosstalk between innate and adaptive immunity. These interactions are important for elucidating the cellular mechanisms that govern immune responses and are beneficial for developing potent immunotherapies.

Numerous studies have found that modulating connexins offers a unique advantage in influencing innate immunity, with broad implications for managing infections, inflammatory conditions, and cancers (99). As a result, understanding the intricacies of this aspect of the immune system opens a new avenue for immune regulation. Researches on drugs targeting connexins reveal their potential to initiate and resolve inflammation as a promising avenue for developing innovative anti-inflammatory strategies. Most drug development programs aimed at modulating gap junctions for therapeutic purposes have focused on Cx43 (124) (**Table 1**). Moreover, connexin hemichannels serve as docking sites for extracellular vesicles, providing new insights for drug delivery (124). Noticeably, manipulating gap junctions may be key to restoring immune tolerance in autoimmune diseases. In summary, the intricate interplay between gap junctions and the innate immune system represents a fast-growing area of research with profound implications for human health. The challenge lies in deciphering the complexities of connexin-mediated immune responses and translating this knowledge into clinical applications, which may ultimately offer innovative therapeutic strategies for disease management.

**Table 1:** Recent Cx43-targeting agents in clinical trials.

Agent	Type	Mode of action	Clinical trial
AsODN	Antisense oligonucleotides	Decrease Cx43 levels	Phase 2
aCT1	Peptide mimetics	Decrease Cx43-ZO-1 interaction	Phase 3
Rotagaptide	Modified peptide	Enhance the gap junction function	Phase 2
Danagaptide	Modified peptide	Enhance the gap junction function	Phase 2
ZP1609	Modified peptide	Enhance the gap junction function	Phase 2
Excleningen	Small molecule compounds	Selectively open Cx43 hemichannels	Phase 1
Carbenoxolone	Small molecule compounds	Decrease Cx43 levels	Orphan drug designation from the FDA
Peptide 5	Peptide mimetics	Decrease hemichannels	Planned
